# 循环肿瘤DNA在淋巴瘤中的进展和应用

**DOI:** 10.3760/cma.j.cn121090-20240528-00197

**Published:** 2024-09

**Authors:** 德智 黄, 曦 张, 军 饶

**Affiliations:** 1 陆军军医大学第二附属医院血液病医学中心，全军临床重点专科，创伤与化学中毒国家重点实验室，重庆市医学重点学科，血液病与微环境重庆市重点实验室，重庆 400037 Medical Center of Hematology, Xinqiao Hospital of Army Medical University, Chongqing Key Laboratory of Hematology and Microenvironment, State Key Laboratory of Trauma and Chemical Poisoning, Chongqing 400037, China; 2 金凤实验室，重庆 401329 Jinfeng Laboratory, Chongqing 401329, China

## Abstract

淋巴瘤是一种多亚型和强异质性的肿瘤，其分子谱分析的金标准方法是基于侵入性的淋巴结或组织活检，但该方法无法准确捕获每个患者体内的空间肿瘤异质性以及全身的肿瘤侵犯和肿瘤负荷情况。循环肿瘤DNA（circulating tumor DNA，ctDNA）是一种新兴且高度通用的生物标志物，它克服了成像扫描和组织活检的基本局限性，具有操作简便快速、无创、特异性好和灵敏度高等特征。ctDNA检测已经应用于淋巴瘤的多种亚型之中，并已用于体细胞突变基因分型、疗效监测、检测微小残留病以及预测预后生存，这些应用有助于临床医师在对淋巴瘤患者诊治时做出更好的临床决策。本文综述了ctDNA分析的不同方法，并描述了ctDNA在不同淋巴瘤亚型中的具体应用。

## Abstract (en)

Lymphomas are a highly heterogeneous group of tumors that are classified into several subtypes. The gold standard method for the molecular profiling of lymphoma is based on invasive lymph node or tissue biopsy. However, this method cannot accurately capture spatial tumor heterogeneity in each patient as well as systemic tumor invasion and tumor burden. Circulating tumor DNA（ctDNA）is an emerging and highly versatile biomarker that overcomes the basic limitations of imaging scanning and tissue biopsy; has the characteristics of being simple, rapid, and non-invasive; and has good specificity and high sensitivity. ctDNA testing has been applied to a variety of subtypes of lymphoma and has been used for somatic mutation genotyping, efficacy monitoring during treatment, detection of minimal residual disease, and the prediction of survival, which may help clinicians make better clinical decisions in the diagnosis and treatment of lymphoma patients. Furthermore, this study also aims to review the different methods of ctDNA analysis and describe the specific applications of ctDNA in different lymphoma subtypes.

淋巴瘤是全球十种最常见的癌症类型之一[1]。世界卫生组织（WHO）于2022年发布的最新国际淋巴瘤亚型分类中，共认定了125种淋巴瘤实体和亚型，准确诊断淋巴瘤及其亚型和评估淋巴瘤肿瘤负荷对于为淋巴瘤患者提供最佳治疗和护理至关重要[2]。目前淋巴瘤肿瘤分子谱分析的金标准方法是基于肿瘤组织活检。然而，组织活检存在一定的局限性：它是一项有创检查，成本高、程序复杂、需要专门的人员和医疗手术室，患者术后需要较长的恢复时间，这使得组织活检无法用于长期监测，而且组织活检缺乏关于肿瘤空间和时间异质性的信息[3]。此外，在某些淋巴瘤亚型中，活检组织难以获得，在这种情况下，可用的组织学样本通常质量较差，会出现取样相关的伪影，少量的肿瘤样本增加了准确病理诊断的困难[4]–[5]。这种困境使得人们开辟了一种新的诊断途径：液体活检。其中以循环肿瘤DNA（ctDNA）作为对象的研究最多，并且ctDNA检测作为一种非侵入性分析方法，可用于不同淋巴瘤类型辅助早期诊断、疾病监测、治疗反应监测和危险度分层等[6]。本文将从ctDNA概念、检测方法和在淋巴瘤中的应用进展等几个方面进行综述。

一、ctDNA概述

循环游离DNA（cell-free DNA，cfDNA）是指在细胞凋亡、增殖和坏死的正常生理过程后自由释放到外周血中的循环双链DNA片段[7]。在肿瘤患者中，来源于肿瘤细胞凋亡、坏死或分泌的cfDNA被称为ctDNA，从血液或其他体液中提取的ctDNA平均浓度可高达180 ng/ml[3],[8]。ctDNA片段长一般为90～150 bp[9]。不同淋巴瘤亚型的cfDNA中ctDNA的含量差异很大，范围为0.1％～95％[10]。与许多基于血液的传统生物标志物相比，ctDNA的半衰期很短，仅为16 min～5 h，作为肿瘤负荷的动态生物标志物，使ctDNA能够实时监测肿瘤的发生和发展情况[11]–[12]。此外，ctDNA作为肿瘤生物标志物的灵敏度和特异性要更佳，并能描述个体间存在较大差异的肿瘤特异性遗传和表观遗传异常，如点突变、结构变异、拷贝数变异、微卫星改变、开放染色质区（open chromatine regions，OCR）和甲基化改变等，这与精准诊疗理念相符，使ctDNA的潜在临床价值远远超出了传统生物标志物[13]–[14]。

虽然ctDNA有诸多的优点和较高的临床应用价值，但由于它绝对含量低、占cfDNA总量少、半衰期短等特点，要将ctDNA应用于临床还需要克服一系列技术上的问题，尤其需要具有灵敏度高和特异性好的突变检测方法，才能够从大量的野生型DNA中扩增并检测出微量的突变型ctDNA[15]。通常，液体活检测定可分为基于聚合酶链式反应（PCR）的方法和基于二代测序（NGS）的方法[16]。基于PCR的靶向方法涉及扩增感兴趣的特定目标DNA序列。这类包括等位基因特异性PCR[17]、数字PCR[18]、微滴数字PCR（ddPCR）[19]和数字PCR-流式技术（BEAMing）[20]等技术，可以检测预先指定的癌症相关突变基因中的突变[18]。NGS是一种强大的高通量测序技术，可快速进行DNA测序并生成大量数据，无需对原发肿瘤进行测序即可同时检测ctDNA的基因组和多种罕见突变，包括靶向测序和非靶向测序两类。靶向测序包括标记扩增子深度测序[21]、安全测序系统[22]和肿瘤个性化深度测序（cancer personalized profiling by deep sequencing，CAPP-Seq）等[23]–[24]。非靶向技术有全基因组测序、全外显子组测序（whole-exome sequencing，WES）和全基因组亚硫酸氢盐测序技术等[14]。

二、ctDNA在淋巴瘤中的应用

（一）ctDNA在淋巴瘤中基因突变检测的应用

Camus等[25]对30例原发性纵隔B细胞淋巴瘤（primary mediastinal B-cell lymphoma，PMBL）患者配对的肿瘤组织DNA和血浆样本ctDNA基因图谱的研究发现，在19例（63.3％）患者中，两者的相似性≥80％。将PMBL的ctDNA特征与经典霍奇金淋巴瘤（cHL）患者队列（60例）的特征进行了比较，两者突变频率排名前3的突变基因均为SOCS1、TNFAIP3和B2M。PMBL在TNFAIP3（71.9％对46.3％，*P*＝0.029）和GNA13（46.9％对17.1％，*P*＝0.013）中显示出比cHL更多的改变。Kim等[26]设计了112个基因组成的panel检测不同类型淋巴瘤以反映它们各自的组织和临床特征，42例患者ctDNA总检出率为85.7％（36/42），高频突变的基因包括PIM1、TET2、BCL2、KMT2D、KLHL6、HIST1H1E和IRF8。其中对18例患者的匹配的组织样本进行NGS分析，在大多数匹配的组织活检样本（88.9％）和血浆样本（83.3％）中至少检测到1种突变，在匹配的血浆样本中也发现了大量在组织样本中检测到的突变（40.4％）。Zhao等[27]设计了59个基因构成的panel，对28例初诊滤泡性淋巴瘤（FL）患者的血浆采用基于NGS的ctDNA检测方法进行了靶向检测来评估突变谱，其中21例患者检测到ctDNA突变。突变频率>10％的突变基因依次有CREBBP、KMT2D、STAT6、CARD11、PCLO、EP300、BCL2和TNFAIP3。KMT2D、EP300和STAT6突变与较差的无进展生存（PFS）相关。此外，ctDNA的变异等位基因频率与FL患者肿瘤负荷相关。

（二）ctDNA在淋巴瘤早期诊断中的应用

Hattori等[28]对14例原发性中枢神经系统淋巴瘤（primary central nervous system lymphoma，PCNSL）患者进行了ddPCR和靶向深度测序，结果显示ddPCR可以灵敏地检测cfDNA中的MYD88^L265P^突变，这种非侵入性的诊断方式可作为组织活检很好的补充。Yamaguchi等[29]基于微流体热循环技术的实时PCR开发一种新的快速基因分型系统（GeneSoC），作者使用GeneSoC对24例PCNSL患者进行了MYD88^L265P^突变检测，结果显示无假阴性。另一项研究对中枢神经系统淋巴瘤（CNSL）患者治疗前的脑脊液（CSF）和血浆进行了ctDNA检测，并开发了一种利用ctDNA鉴定CNSL的机器学习方法，其在CSF和血浆样本中分别显示出59％和25％的敏感性，具有较高的阳性预测价值[30]。Hayashida等[31]对20例血管免疫母细胞性T细胞淋巴瘤患者进行cfDNA检测，发现分别有14例和3例患者的cfDNA中分别携带RHOA^G17V^和IDH2^R172^突变。由于IDH2^R172^突变的频率低于RHOA^G17V^，并且与后者突变共存，因此，RHOA^G17V^突变的检测更具有诊断价值。

（三）ctDNA在淋巴瘤分型中的应用

Alig等[32]对366例cHL患者的治疗前血浆样本通过CAPP-Seq和（或）WES进行了分析，用聚类方法揭示两种不同的cHL基因组亚型：H1和H2。它们具有特征性的临床和预后相关性，以及不同的转录和免疫特征。作者在外部数据集中验证了两种亚型的遗传类别，并通过非侵入性转录谱分析以及血浆和肿瘤组织的免疫学谱分析等正交方法显示其独特的生物学特征。综合正交观察结果，H1亚型似乎更多地受到NF-κB信号传导、cHL肿瘤微环境内的串扰信号传导、细胞因子受体和下游STAT信号传导的驱动。这种精氨酸驱动的表型与H1亚型中MHC Ⅰ类分子的频繁丢失一起作为肿瘤免疫逃逸机制。相比之下，H2亚型似乎是由更“常规”的淋巴瘤驱动因子，如KMT2D和TP53以及类似于弥漫大B细胞淋巴瘤（DLBCL）中A53亚型的遗传不稳定性驱动的，这些体细胞变化还与其他机制配合，包括驱动T细胞耗竭的CD274扩增。

（四）ctDNA在检测微小残留病（MRD）和疗效监测研究中的应用

Scherer等[33]使用CAPP-Seq技术追踪41例DLBCL患者ctDNA中的多个体细胞突变，从第1个ctDNA阳性时间点到临床复发之间的平均间隔时间为188 d，复发前3个月内采集的所有血液样本中ctDNA的浓度都超过了检测方法的检测限。与高通量测序的克隆型免疫球蛋白重链基因（IgHTS）技术比较，基于CAPP-Seq的ctDNA检测到的MRD阳性患者数量是IgHTS的两倍，平均前导时间大于2个月，这表明该方法在疾病监测方面具有潜在优势。Heger等[34]基于ddPCR对12例复发/难治性（R/R）DLBCL患者进行了ctDNA监测，该研究也证实了基于ddPCR从血浆来源的ctDNA中检测患者特异性V（D）J序列是可行的，并且该方法价格实惠、周转快，可用来评估MRD，对R/R DLBCL的结局具有高度预测性。在另一项ctDNA连续监测8例CD19靶向的CAR-T细胞治疗R/R DLBCL患者的研究中，与PET-CT相比，ctDNA分析检测肿瘤相关ctDNA突变的灵敏度和特异性分别为94.7％和83.3％，GNA13、SOCS1、TNFAIP3和XPO1突变与CAR-T细胞治疗的不良预后相关，显示出ctDNA检测在CAR-T细胞治疗监测MRD、揭示预后、对患者进行分层和指导精确治疗中的巨大潜力。Dean等[35]对57例接受阿基仑赛治疗的R/R大B细胞淋巴瘤（LBCL）患者使用ctDNA检测和PET-CT扫描以比较两者疗效监测的效果，结果显示ctDNA和肿瘤代谢体积在治疗1个月时没有相关性（*P*＝0.110），但在3个月时相关（*P*<0.001），这表明接受CAR-T细胞治疗的R/R LBCL患者在治疗1个月后，进行血浆ctDNA检测可作为标准PET-CT扫描成像有价值的补充检测，两者联合应用可提高疗效监测的准确性。

Kim等[36]对94例外周T细胞淋巴瘤患者的血浆cfDNA进行靶向测序后，发现53例（56.4％）患者检测到血浆ctDNA突变，66个测序基因中有51个检测到体细胞突变，排名前10的基因有RHOA、CREBBP、KMT2D、TP53、IDH2、ALK、MEF2B、SOCS1，CARD11和KRAS。治疗结束时与基线相比，基因组当量（genomic equivalent，GE）降低≥1.5 log的患者比降低<1.5 log患者有更好的生存结果。Lakhotia等[37]发现在套细胞淋巴瘤中，在2个周期的诱导治疗后ctDNA阴性的患者比ctDNA阳性的患者具有更长的PFS期和总生存（OS）期。所以，早期清除ctDNA的患者的预后相对较好，这为确定是否需要进行积极的巩固治疗提供了有价值的工具，并可作为制定风险适应的治疗策略的依据。

（五）ctDNA在淋巴瘤患者生存预后中的应用

Le Goff等[38]对112例初诊LBCL用NGS的方法检测ctDNA，ctDNA浓度>3.57 log hGE/ml与患者较低的1年PFS率相关（44％对83％，*P*<0.001）。另一项针对101例初诊高危DLBCL的ctDNA检测显示，ctDNA浓度>3.75 log hGE/ml患者OS明显更差（*P*＝0.0024），且ctDNA浓度是OS的独立预后因素[39]。Herrera等[40]对接受苯达莫司汀和利妥昔单抗（联用或不联用维泊妥珠单抗）的R/R DLBCL患者进行ctDNA测量，高ctDNA浓度患者PFS更差。一项针对23例接受CD19靶向CAR-T细胞治疗的R/R LBCL患者的ctDNA监测发现，在接受CAR-T细胞治疗的LBCL患者中，首次报道了较短的ctDNA片段（<170 bp）与较差的PFS和OS显著相关[41]。

近期，Mutter等[30]对92例CNSL患者治疗前血浆中采用了基于CAPP-Seq的ctDNA方法进行了检测，ctDNA阳性的患者PFS期和OS期显著缩短，治疗前血浆ctDNA浓度是预后的独立影响因素。在治愈性免疫化疗期间通过对患者血浆ctDNA监测发现，有可测量的残余疾病检出的患者预后特别差。该研究结果强调了ctDNA作为一种非侵入性生物标志物的作用及其在CNSL患者个性化风险分层和治疗指导中的潜在价值。更进一步，Heger等[42]将超灵敏ctDNA测序应用于67例CNSL患者的146份血浆和CSF样本，追踪了血浆来源ctDNA中的肿瘤特异性突变，并基于此开发了一种新的CNSL生物标志物：外周残余疾病（peripheral residual disease，PRD）。治疗后PRD的持续性检出是复发的高度预测因素。将已建立的基线临床风险因素与治疗期间的放射学反应和PRD评估相结合，作者开发并独立验证了一种新的风险分层工具：CNSL分子预后指数（molecular prognostic index for CNSL，MOP-C）。MOP-C被证明对CNSL患者的预后具有高度预测性。MOP-C是结合CNSL临床风险因素和分子特征的完全无创的动态风险监测模型，该模型有可能提高目前CNSL患者的临床风险分层和放射学反应评估标准，最终为个性化治疗铺平道路。

本课题组前期发现结外NK/T细胞淋巴瘤（ENKTL）患者中最常发生突变的基因是KMT2D（23.1％），且KMT2D与ENKTL患者预后呈负相关[43]。我们进一步发现高ctDNA浓度（>4.83 log hGE/ml）与较差预后显著相关，将ctDNA浓度与带有EBV-DNA的自然杀伤淋巴瘤预后指数（prognostic index of natural killer lymphoma with EBV-DNA，PINK-E）相结合构建PINK-EC预后模型，PINK-EC能有效地把初诊ENKTL患者区分为低、中、高三个风险分层，并且预后预测和风险分层效能均优于国际预后指数（IPI）、韩国预后指数（KPI）和PINK-E[44]。Tian等[45]通过LASSO-Cox得到ctDNA甲基化预后（ctDNA methylation prognosis，cmp）分数，把cmp分数与PINK整合构建PINK-C预后预测模型，显示了良好的预后预测和风险分层结果。Kim等[46]发现在ENKTL治疗前样本中有DDX3X突变的患者预后差。总体而言，ctDNA的基因突变、浓度和甲基化模式等变化可以为预测ENKTL患者的预后提供参考。

三、ctDNA检测的局限与展望

当前，淋巴瘤治疗中的临床决策越来越多地由分子分层来定义，众多淋巴瘤亚型中特定基因组生物标志物的确定推动了靶向治疗和其他系统疗法的发展。灵活的诊断方法可以指导医师为患者在众多的治疗手段中采取最佳的疗法。目前，虽然ctDNA检测在临床上的用途仅限于辅助治疗选择，但在不久的将来，定期对患者进行纵向分析并捕捉ctDNA动态变化将为淋巴瘤患者的治疗和护理提供更多的帮助和指导。所以，我们认为下一个关键应用领域是疗效评估和耐药性监测。根据我们的设想，纵向ctDNA监测将比影像学检测更灵活地识别治疗反应或疾病进展，增强对模棱两可的扫描结果的解释，从而更及时地改变对个别患者的系统治疗。而这也可有助于患者产生耐药时在经历临床确认的淋巴瘤进展之前对治疗方案进行修改。总的来讲，ctDNA有可能作为一种新的替代终点，帮助加速和改善临床试验设计和药物开发[5]。

尽管ctDNA在淋巴瘤中应用前景广阔，但仍有一些关键问题需要在未来优先解决。首先，ctDNA分析的预处理，如采血管的选择和离心等需要进行优化和标准化以确保可靠的检测结果。其次，血浆ctDNA的浓度通常相对较低，ctDNA检测的灵敏度仍有待提高。再其次，需要更多的试验来寻找淋巴瘤的特异性ctDNA生物标志物，提高早期筛查的检出率。此外，开发新的检测方法或结合不同的高通量测序及其他检测方法来提高淋巴瘤ctDNA的检出率也迫在眉睫。更重要的是，ctDNA的含量受到肿瘤分期、异质性和克隆性等因素的影响。因此，有必要建立统一的ctDNA检测方法和检测时间标准，从而更方便、准确地分析ctDNA在不同类型、分期的淋巴瘤临床治疗中的相关性，为肿瘤监测和治疗提供明确的指导。

总之，作为一种新型非侵入性肿瘤生物标志物，ctDNA为淋巴瘤在突变分析、诊断、疗效监测和预后预测等方面带来了显著变化。ctDNA的发展有可能加深我们对癌症生物学的理解，并在疾病早期阶段提供有价值的信息，指导未来淋巴瘤风险适应性和基于机制的治疗以及实现更高水平的个性化治疗。
